# Clinical Utility of an Alzheimer’s Disease Blood Test Among Cognitively Impaired Patients: Results from the Quality Improvement PrecivityAD2 (QUIP II) Clinician Survey Study

**DOI:** 10.3390/diagnostics15020167

**Published:** 2025-01-13

**Authors:** Mark Monane, Demetrius M. Maraganore, Robert M. Carlile, Kim G. Johnson, David A. Merrill, Darren R. Gitelman, Kenneth S. Sharlin, Lawren A. VandeVrede, Kristi K. George, Jimin Wang, Tim West, Leslie Jacobs, Philip B. Verghese, Joel B. Braunstein

**Affiliations:** 1 C_2_N Diagnostics, LLC, 4340 Duncan Avenue, St. Louis, MO 63110, USA; twest@c2n.com (T.W.); ljacobs@c2n.com (L.J.); pverghese@c2n.com (P.B.V.); jbraun@c2n.com (J.B.B.); 2 Department of Neurology, Tulane University School of Medicine, New Orleans, LA 70112, USA; 3 Palmetto Primary Care Physicians, Summerville, SC 29486, USA; rmcarlile@palmettoprimarycare.com; 4 Department of Neurology, Duke University School of Medicine, Durham, NC 27710, USA; kim.g.johnson@duke.edu; 5 Pacific Neuroscience Institute, Santa Monica, CA 90404, USA; dmerrill@pacificneuro.org; 6 Advocate Lutheran General Hospital, Park Ridge, IL 60068, USA; 7 Sharlin Health and Neurology, Ozark, MO 65721, USA; drsharlin@sharlinfxmed.com; 8 UCSF Weill Institute for Neurosciences, San Francisco, CA 94103, USA; lawren.vandevrede@ucsf.edu; 9 JWM Neurology, Indianapolis, IN 46256, USA; 10 Stat4ward, Pittsburgh, PA 15238, USA; bob.wang@stat4ward.com

**Keywords:** Alzheimer’s disease, blood biomarker, diagnosis, clinical decision-making, clinical utility, memory care specialists, mild cognitive impairment, dementia

## Abstract

**Objective**: The objective of this study was to assess clinical decision-making associated with the use of a multi-analyte blood biomarker (BBM) test among patients presenting with signs or symptoms of mild cognitive impairment or dementia. **Methods**: The Quality Improvement PrecivityAD2 (QUIP II) Clinician Survey (NCT06025877) study evaluated the clinical utility of the PrecivityAD2™ blood test in a prospective, single cohort of 203 patients presenting with symptoms of Alzheimer’s disease (AD) or other causes of cognitive decline across 12 memory specialists. The PrecivityAD2 blood test (C2N Diagnostics, St. Louis, MO) combines the plasma Aβ42/Aβ40 ratio and the p-tau217/np-tau217 ratio (%p-tau217) measurements in a statistical algorithm to yield an Amyloid Probability Score 2 (APS2) that informs on the likelihood of brain amyloid plaques. After receiving the BBM test results, clinicians completed surveys on management strategies for each patient. **Results**: Patients had a median age of 74, 53% were female, and 28% were traditionally under-represented in Black, Hispanic, and Asian groups. The composite primary endpoint, defined as a change in AD diagnostic certainty, drug therapy, or additional brain amyloid evaluation pre- and post-BBM testing, was 75% (*p* < 0.0001 versus the pre-specified threshold of 20% clinically meaningful change). Anti-AD medication orders decreased among negative APS2 patients and increased among positive APS2 patients (*p* < 0.0001). Additional brain amyloid testing decreased among negative APS2 patients (*p* < 0.0001). **Conclusions:** This blood biomarker test can help memory specialists guide patients to anti-AD therapies as well as rule out AD to allow for other diagnostic considerations.

## 1. Introduction

Of the more than 100 million adults in the US over 55, ~9 million have mild cognitive impairment (MCI), and ~7 million have dementia [1,2]. These individuals warrant a diagnostic evaluation to determine the cause of their signs or symptoms of cognitive decline. Alzheimer’s disease (AD) is the underlying cause in 30% and 60% of MCI and dementia cases, respectively, and the prevalence of AD increases from 14% to 35% with increasing age over 60 years old [3,4,5].

Traditionally available AD pathology tests for diagnostic evaluation of brain amyloid include imaging with amyloid positron emission tomography (PET) scan or cerebrospinal fluid (CSF) biomarker analysis through a lumbar puncture. Although well-established and accepted as valid clinical diagnostic testing options, each has access, ease-of-use, and other limitations. Amyloid PET scan usage is associated with radiation and patient reluctance, susceptibility to reader bias, and challenges in healthcare equity and inclusion [6,7]. CSF analysis is invasive, time-consuming, contraindicated in up to 15% of individuals (due to bleeding risk), associated with adverse events, unappealing to many individuals, and may pose access challenges, particularly among minority and other underserved populations [8].

The clinical integration of blood biomarkers (BBMs) for AD holds promise in enabling the early detection of pathology and timely intervention [9]. The use of a blood biomarker test that is scalable and accessible as well as acceptable and equitable may address the unmet need in diagnostic testing [10,11]. The recent Food and Drug Administration approvals of two disease-modifying treatments (DMTs) for MCI and dementia are marking the beginning of a new era for therapeutic development and clinical management plans for patients experiencing MCI or dementia due to AD [12]. Because these DMTs target aggregated soluble and insoluble forms of amyloid beta (Aβ) protein, biomarker confirmation of amyloid pathology is necessary before initiation. The success of these new treatment options will likely rely on strategies for identifying eligible patients with brain amyloid pathology and evaluating their DMT benefit-to-risk profile, including a biomarker-guided algorithm for early and accurate diagnosis [8].

In 2020, the first commercially available blood test for AD pathology, the PrecivityAD^®^ blood test (which included Aβ42/40, apolipoprotein E, and age), was released and showed strong clinical validity [13] in identifying brain amyloid status when compared to amyloid PET as well as robust clinical utility [14]. Building on this experience and incorporating phosphorylated tau that has been shown to exhibit high accuracy for distinguishing AD from other neurodegenerative diseases in patients with cognitive impairment [15], the PrecivityAD2™ blood test (C2N Diagnostics, LLC, St. Louis, MO, USA), a high-resolution mass spectrometry-based test with an algorithm combining %p-tau217 and Aβ42/40 ratio to identify presence of brain amyloid, was designed to aid healthcare providers in ruling in or ruling out AD in cognitively symptomatic patients. The PrecivityAD2 blood test is intended for use in patients aged 55 and older with signs or symptoms of MCI or dementia who are undergoing evaluation for AD or other forms of cognitive decline.

The analytical validity [16] and clinical validity [17,18] of this multi-analyte BBM test have been established with robust test performance and high accuracy in the intended use population. In a study of 583 patients, the overall percent agreement with PET scan results was 88% in a population with a 53% prevalence of amyloid positivity [17]. Among 1213 patients in primary care and secondary care undergoing clinical evaluation due to cognitive symptoms, the diagnostic accuracy of the test was 90% using CSF analysis as the reference standard [18]. However, evidence on how high-performing BBMs can best be used in clinical settings to optimize AD clinical decision-making is still emerging [19]. The objectives of this study were to assess clinicians’ concordance with the intended use of this multi-analyte blood biomarker in their clinical patients, as well as the effect of the test and test result on clinical decision-making, including diagnostic certainty that informs appropriate medication changes and additional test ordering.

## 2. Materials and Methods

### 2.1. Study Subjects

Study patients who were 55 years of age and older presenting to participating sites with signs or symptoms of MCI or dementia and undergoing evaluation for AD or other causes of cognitive decline were eligible for study inclusion. Patients who were being assessed for brain amyloid as a cause of their symptoms were included in this study. Patients who had other likely non-AD-related causes for their cognitive impairment were not included.

### 2.2. Study Sites

Participating sites were representative of private practices and university-affiliated memory and dementia clinics across the United States. Up to two clinicians per site participated in this study. Participating clinicians included neurologists, geriatricians, and geropsychiatrists, as well as other licensed healthcare providers in their offices who evaluate patients presenting with signs or symptoms of MCI or dementia.

### 2.3. Study Design

#### 2.3.1. Study Tool

The PrecivityAD2 blood test quantifies specific plasma amyloid beta and tau peptide concentrations using immunoprecipitation followed by liquid chromatography–tandem mass spectrometry (LC-MS/MS). The technology simultaneously quantifies amyloid beta 42 (Aβ42) and Aβ40 peptide isoform concentrations and phosphorylated and non-phosphorylated tau at amino acid threonine, position 217 (p-tau217 and np-tau217, respectively), peptide concentrations. The Aβ42/40 ratio and the percent p-tau217 phosphorylation (p-tau217/np-tau217, %p-tau217) are calculated from the concentrations of the peptides.

A logistic regression algorithm combines Aβ42/40 ratio and %p-tau217 measurements to generate the Amyloid Probability Score 2 (APS2), a numerical value ranging from 0 to 100, which indicates the likelihood of the presence of brain amyloid plaques as detected by amyloid PET scan. A negative APS2 (0–47) result is consistent with a negative amyloid PET scan and, therefore, is not consistent with a neuropathological diagnosis of AD. A positive APS2 result (48–100) is consistent with a positive amyloid PET scan and, therefore, is consistent with a neuropathological diagnosis of AD. The performance of this BBM test has been validated as follows: accuracy was 88% (95% CI: 85 to 91%), sensitivity was 88% (95% CI: 84–91%), specificity was 89% (95% CI: 84–92%), positive predictive value was 90% (95% CI: 86–93%), and negative predictive value was 87% (95% CI: 82–90%) [17].

Similar to PET and CSF biomarker tests for AD, this BBM test is not designed as a standalone diagnostic test and is clinically available for patients when ordered by healthcare providers as part of the evaluation for AD and interpreted in the context of the patient’s medical information. This laboratory-developed test is commercially available in 49 US states (pending in New York), the District of Columbia, and Puerto Rico.

#### 2.3.2. Study Conduct

The Quality Improvement and Clinical Utility PrecivityAD2 Clinician Survey (QUIP II) study was a single-arm, multi-site prospective cohort study that assessed the association between the PrecivityAD2 blood test and subsequent changes in clinical work-up and management. The memory specialists received education and training on the intended use of this BBM test as well as the APS2 result. Plasma collection and shipping were performed in accordance with C2N’s standard methods. Briefly, EDTA blood specimens were collected from participating study subjects, centrifuged, and plasma aliquoted prior to shipping. Samples were shipped on the day of collection using a refrigerated shipping solution to the C2N CAP (College of American Pathologists)-accredited, CLIA (Clinical Laboratory Improvements Amendments)-certified laboratory for analysis. The C2N laboratory performed the test according to validated procedures, with test results returned to the ordering study clinician by dedicated fax line.

Each clinician then completed a survey that was built within a HIPAA (Health Insurance Portability and Accountability Act) compliant survey system (SurveyMonkey^®^, SurveyMonkey, Inc., San Mateo, CA, USA). The survey collected patient demographics, clinician information, and feedback concerning pre- and post-BBM test diagnostic certainty, as well as pre- and post-BBM test patient management plans, including medication prescribing and additional brain amyloid evaluation. APS2 results were interpreted by clinicians at their own discretion.

This study was reviewed and found to be exempt from institutional review board (IRB) oversight by a national IRB (Advarra, Inc., Columbia, MD). The protocol was submitted to central and local IRBs in alignment with institutional policies. All IRBs are granted an exemption from oversight. This study followed the CONSORT/STROBE reporting guideline framework [20]. The QUIP II study is registered on clinicaltrials.gov (NCT06025877).

#### 2.3.3. Primary Outcome

The primary outcome of this study had two parts: patient selection and score interpretation. Patient selection was evaluated in terms of concordance of clinicians’ test ordering with the intended use criteria of the PrecivityAD2 blood test. Clinical decision-making, a measurable proxy for interpretation of the test’s APS2 result, was evaluated in terms of changes in clinician-reported probability of AD (0–100%) pre- and post-BBM testing as well as AD drug therapy (acetylcholinesterase inhibitors, memantine, and lecanemab) and additional amyloid brain evaluation pre- and post-BBM testing as reported on the clinician survey.

#### 2.3.4. Secondary Outcomes

Secondary outcomes included the results of each subpart of the primary outcome. Concordance with age and concordance with symptoms criteria were evaluated. Changes in clinician-reported AD diagnostic certainty were measured pre- and post-BBM testing; changes in medication prescribing and additional test ordering were measured pre- and post-BBM testing.

### 2.4. Statistical Analysis

For the intended use component as part of the primary outcome, concordance was measured and compared to the benchmark of 100% using Fisher’s exact test. That is, the concordance was calculated as $Concordance_{IU}=N_{1}/N$ where $N_{1}$ is the number of patients aged 55 years and older with signs or symptoms of MCI or dementia suggestive of Alzheimer’s disease, and N is the total number of enrolled subjects. The $Concordance_{IU}$ was compared to the benchmark of 100% using Fisher’s exact test.

For the composite endpoint on clinical decision-making, the percentage of change in composite endpoint was measured by the percentage of enrolled subjects showing change in composite endpoint. The percent was calculated as $\%Change_{\mathrm{Composite}\;\mathrm{Endpoint}}=N_{1}/N$, where $N_{1}$ is the number of patients with any change in medication or in testing or with clinician-reported probability of AD changing across the 50% threshold (i.e., from ≤50 to >50), from pre- to post-BBM test, and N is the total number of enrolled subjects. To assess clinically significant changes in decision-making associated with BBM testing, the $\%Change_{\mathrm{Composite}\;\mathrm{Endpoint}}$ was compared to a pre-specified benchmark of 20% relative change using Chi-squared test [21].

In addition, unconditional logistic regression was employed to assess the likelihood of change versus no change in decision-making, with odds ratios calculated from the output generated using the generalized linear model (GLM Function) in R regression [22]. This analysis examined several potential determinants of changes in decision-making, including age, sex, race, ethnicity, pre-test probability of disease, pre-test diagnosis, APS2 result, and study site. This approach facilitated the evaluation of the contributions of each variable to the outcome. Confidence intervals for the estimated odds ratios and significance tests for differences from the null value were calculated using the estimated standard errors [23]. Two-tailed *p*-values less than 0.05 were considered statistically significant.

Additional secondary measures were calculated, including concordance with age or diagnosis and the percentage of change in AD diagnostic certainty. The distributions of clinician-reported probabilities of Alzheimer’s disease (AD) pre- and post-BBM testing were compared using the Kolmogorov–Smirnov (KS) test. The correlation between APS2 and clinician-reported probability of AD was compared using a scatterplot and fitted using linear models. Pre-test clinician-reported probability of AD was compared by degree of cognitive impairment, by clinician type, and by site. The change in AD drug prescribing and additional brain amyloid evaluation were also reported by APS2 results. The number and percentage of patients with each type of medication use and additional brain amyloid testing ordered pre- and post-BBM test were reported and compared using the hypothesis test method using Chi-squared test.

All hypothesis testing was 2-sided, and a *p*-value < 0.05 was considered statistically significant. All data analyses were performed using R 4.2. software [24].

## 3. Results

### 3.1. Study Participants

#### 3.1.1. Patients

A total of 213 BBM tests were performed on 203 patients in the study cohort from November 2023 to May 2024: 10 samples were outside the sample acceptance criteria and were rejected, and 203 test reports (95%) were returned to the clinicians. The median age of this final analysis cohort was 74, 53% were female, and 28% were identified by the clinician as typically under-represented Black, Hispanic, and Asian minorities (Table 1).

#### 3.1.2. Memory Specialists

A total of 12 memory specialists, comprising 8 neurologists and 4 other memory care specialists (geriatricians, geropsychiatrists, others) from eight sites (three academic medical centers and five private medical group practices) were included. Geographic distribution of participating sites by US census regions included two sites in the West, three sites in the Midwest, and three sites in the South.

### 3.2. PrecivityAD2 Blood Test Results (APS2)

APS2 results were returned to clinicians with a median turnaround time of 6 business days from the date of specimen receipt by the laboratory. The median APS2 for the overall cohort was 52, with a range of 0–100. Using the pre-specified APS2 result cutpoint, 51% (104/203) of patients had a negative test result, and 49% (99/203) of patients had a positive test result.

### 3.3. Primary Outcome

**Figure 1:**
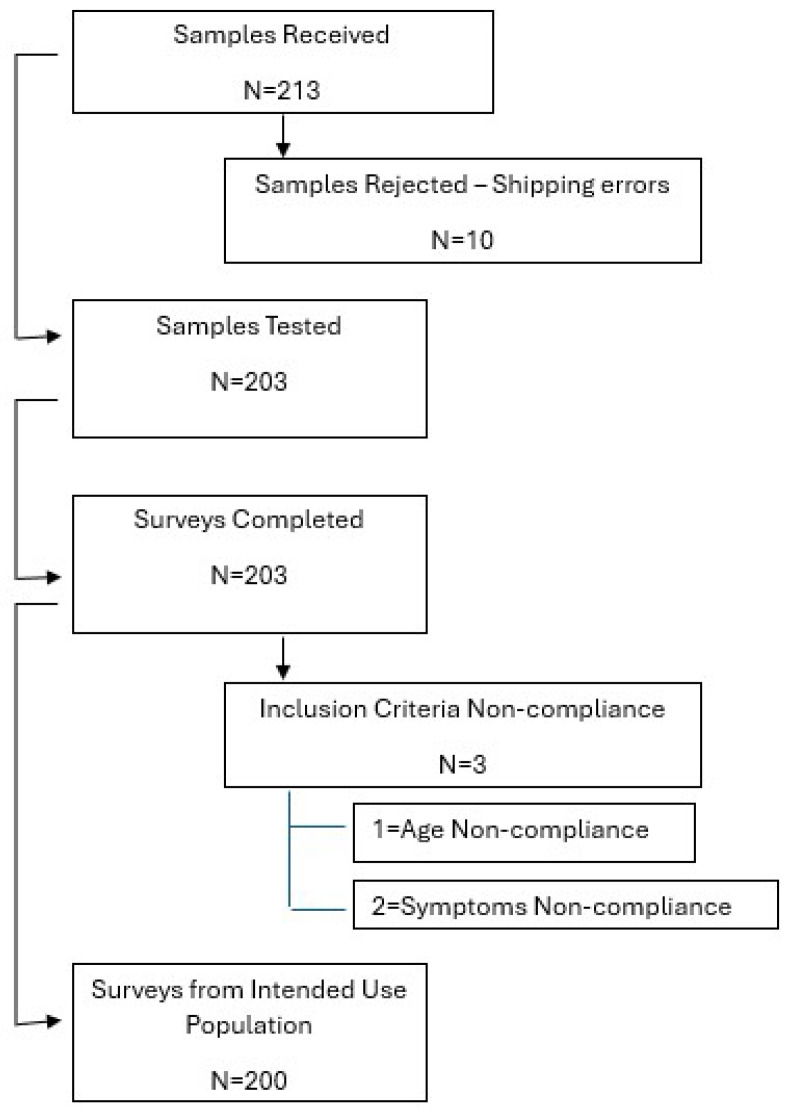
CONSORT/STROBE diagram with patient blood sample and clinician survey flow. Legend for Figure 1: A total of 213 patient blood samples were received, and 10/213 (6%) were not evaluable. A total of 203 patients were included in the final analysis: 200/203 (99%) patients met the intended use criteria for the test. Clinicians completed 203/203 (100%) surveys matched to these patient blood samples.

Concordance with the intended use of the BBM test was 99% (200/203). Reasons for non-concordance were test use outside of the intended use, including patients below the age of 55 (*n* = 1) and patients without symptoms of MCI or dementia (*n* = 2) (Figure 1). The composite primary endpoint, defined as a change in AD diagnostic certainty, drug therapy, or additional brain amyloid evaluation pre- and post-BBM testing, was 75% (153/203, *p* < 0.0001 versus a pre-specified threshold of 20% clinically meaningful change). In the logistical model, to ascertain the individual contribution of the study variables on the primary outcome, only the APS2 result was a significant contributor (OR 2.12 for negative APS2 result, *p* < 0.05).

### 3.4. Secondary Outcomes

#### 3.4.1. Clinician-Reported Probability of Alzheimer’s Disease Pre- and Post-BBM Testing

**Figure 2:**
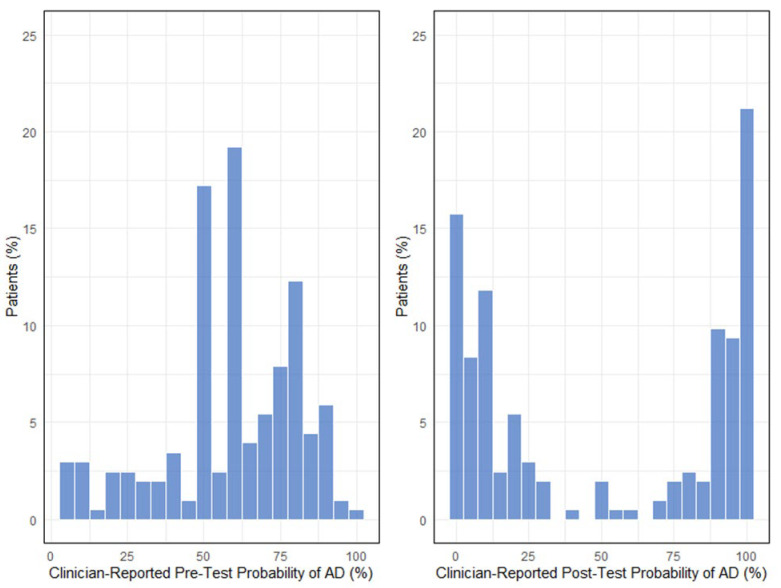
Change in distribution of the clinician-reported probability of Alzheimer’s disease (AD) pre- and post-BBM testing. Legend for Figure 2: Clinician-reported probability of disease is reported in percentage. Pre-test probability (figure on left) was derived from the clinician survey to reflect the probability of AD before BBM testing. Post-BBM test probability (figure on the right) was derived from the clinician survey to reflect the probability of AD after BBM testing. Data were analyzed using the Kolmogorov–Smirnov (KS) Test. The *p*-value is less than 0.001, indicating that the distributions of AD probability (pre- and post-BBM test) are significantly different, suggesting that the two groups yield different results.

Overall, clinician-reported probability of AD diagnosis shifted greatly from pre- to post-BBM testing and became more concentrated in the low and high probability ranges (*p* = 0.005) (Figure 2). On an individual patient level, there was a strong alignment between the changes in clinician-reported probability of AD and the APS2 result. From pre- to post-BBM testing, the mean clinician-reported AD probability decreased from 53% to 11% among negative result patients (*p* < 0.0001) and increased from 65% to 93% among positive result patients (*p* < 0.0001). Changes in clinician-reported probability of AD across the 50% threshold from pre-test to post-BBM test were noted in 38% (77/203) of patients. Comparison testing across the clinical practice study sites and across clinicians at each site did not reveal evidence of heterogeneity for these study outcomes (*p* = 0.22).

The association between APS2 result and clinician-reported probability of AD showed a notable change in pre- to post-BBM testing. Prior to BBM testing, clinician-reported pre-test probability of AD had a weak correlation with APS2 results (Pearson’s correlation coefficient of 0.29, slope of 0.16, *p* < 0.0001). However, after BBM testing, clinician-reported probability of AD had a strong positive correlation with APS2 results (Pearson’s correlation coefficient of 0.95, slope of 1.1, *p* < 0.0001).

#### 3.4.2. Drug Therapy for Alzheimer’s Disease Pre- and Post-BBM Testing

Overall, 59% (119/203) of patients had planned changes in their AD drug therapy (*p* < 0.0001 as compared to the pre-specified benchmark of 20% change). The changes in AD drug therapy on an individual patient basis were aligned directionally with the APS2 results (Table 2). Among patients with a negative test result, the overall use of AD drug therapy decreased significantly pre- to post-BBM testing from 74% (77/104) to 19% (20/104), representing a 74% relative decrease (*p* < 0.0001). Among patients with a positive test result, the overall use of AD drug therapy increased significantly pre- to post-BBM testing from 71% (71/99) to 96% (96/99), representing a 35% relative increase (*p* < 0.0001). Specifically, planned changes in the disease-modifying lecanemab use were related to APS2 results, with significantly decreased use among patients with a negative test result and significantly increased use among patients with a positive test result (*p* < 0.02) (Supplementary Table S1).

#### 3.4.3. Additional Brain Amyloid Evaluation for Alzheimer’s Disease Pre- and Post-BBM Testing

Overall, 33% (68/203) of patients had changes in planned additional brain amyloid evaluation (*p* < 0.0001 as compared to the pre-specified benchmark of 20% change), leading to a 50% overall decrease in additional brain amyloid testing (*p* < 0.001). The changes in additional amyloid testing on an individual patient basis were aligned directionally with the APS2 results (Table 3). Among patients with a negative test result, the overall use of additional amyloid testing decreased significantly pre- to post-BBM testing from 41% (43/104) to 13% (13/104), representing a 70% relative decrease (*p* < 0.0001). Among patients with a positive test result, the overall use of additional amyloid testing also decreased from pre- to post-BBM testing from 35% (35/104) to 26% (26/99), representing a 26% relative decrease (*p* = 0.22).

## 4. Discussion

This study shows that the incorporation of the PrecivityAD2 blood test into memory specialist clinical care of patients undergoing evaluation for cognitive impairment was associated with strong concordance with the test’s intended use criteria as well as clinically meaningful changes in clinical decision-making. Specifically, patients with a negative test result were judged by clinicians to have lower AD likelihood post-BBM test and were less likely to be managed with anti-AD drugs and undergo further brain amyloid evaluation, consistent with a care pathway ruling out AD. Conversely, patients with positive test results were judged by clinicians to have higher AD likelihood post-BBM test and were more likely to be managed with anti-AD drugs, consistent with a care pathway ruling in AD. Of note, the distribution of APS2 results seen in this QUIP II study was similar to that observed in the clinical validation studies to date (49% versus 52% Positive results, respectively) [17].

Interestingly, patients with positive test results also had a decrease in additional brain amyloid evaluation after BBM testing, although this difference from pre-testing was not statistically significant. Currently, many commercial payers provide reimbursement coverage for lecanemab using amyloid PET scan or CSF analysis as the required prior authorization criteria and thus do not allow BBM test results as evidence of brain amyloid pathology. This position by commercial payers is likely significantly biased and reduces the magnitude of the observed effect for the change in downstream additional testing observed from the positive test results. In a survey performed after study closure, the study investigators expressed robust consensus (88% with Very Strongly or Strongly Agree ratings) on the lack of clinical need for further brain amyloid assessments after receiving the APS2 result. This potential role of BBM in replacing or substituting PET and CSF biomarkers has been outlined recently in several workgroup recommendations and is outlined in more detail below [25,26].

The EU/US CTAD Task Force in 2022 suggested that blood biomarkers have the potential to be more accessible and lower cost [27]. More recently, in 2024, the Alzheimer’s Association Workgroup report noted that an abnormal blood biomarker result is sufficient to establish a diagnosis of AD, inform clinical decision-making throughout the disease continuum, and increase confidence that AD is contributing to symptoms [28]. Furthermore, the BBM Workgroup convened by the Global CEO Initiative (CEOi) on Alzheimer’s Disease recommendations addressed the area of AD pathology confirmation before DMT use and concluded that (1) elevated brain amyloid can be detected via amyloid PET imaging, CSF analyses, or BBMs, and (2) high-accuracy BBMs (defined as a BBM test with performance equivalent to that of CSF tests—a sensitivity and specificity of ~90%) could replace PET/CSF biomarker testing for many patients [29]. In this clinical care pathway involving a comprehensive work-up by a memory specialist, a positive BBM test result prompts a thorough discussion on the risks and benefits of disease-modifying therapy for patients who meet eligibility criteria. Conversely, a negative BBM test result prompts evaluation and interventions for non-AD causes of patients’ symptoms [10].

The BBM test used in this study has been shown to reach the recommended levels of clinical performance as laid out in the above-outlined CEOi Workgroup Report as a confirmatory blood biomarker test. Furthermore, the 2024 revised criteria from the Alzheimer Association Workgroup include %p-tau217 (one of the multi-analytes in this BBM test) as a Core 1 biomarker that is sufficient to establish a diagnosis of AD and to inform clinical decision-making throughout the disease continuum [28]. However, there was still an unaddressed question on the effective integration of such high-performance BBM tests and their results into clinical care. Importantly, we believe that the QUIP II study results provide information on this next frontier following these recent expert consensus and workshop recommendations—the effect of BBM results on clinical decision-making in a real-world setting.

Given the major capacity constraints and drawbacks of amyloid PET and CSF testing, integration of high-performance BBM tests in clinical care, as described above, provides the path to facilitate an early and accurate diagnosis, which has been associated with improved patient outcomes [30]. In terms of patient satisfaction, patients have a right to know, and a recent survey by the Alzheimer’s Association found that 70% of Americans would want to know early if they have AD to allow for earlier treatment [31,32]. Furthermore, an early and accurate AD diagnosis can facilitate better preparedness of both the patient and their family for what to expect in terms of activities of daily living and changes in mood and behavior [33]. The harmful financial effects of undiagnosed memory disorders exacerbate the already substantial financial pressure households face upon diagnosis of a memory disorder [34]. Most importantly, similar to patterns noted in oncology care, early detection of AD allows for early access and early treatment to allow for improved clinical outcomes: BBM tests could enable more patients to benefit from new DMTs for early symptomatic MCI or early dementia and lead to preserved/improved cognition and enhanced quality of life [35,36,37]. While other recently emerged biomarker tests, including salivary biomarkers [38] and digital biomarkers [39], as well as new diagnostic tools such as machine learning [40], have been utilized in individuals with cognitive decline, none of these diagnostic strategies for the assessment of AD have reached the level of evidence and high performance seen to date with select blood biomarkers.

Importantly, the integration of blood biomarkers into usual care presents an opportunity to improve health equity. The Center for Medicare and Medicaid Services (CMS) defines health equity as the attainment of the highest level of health for all people, where everyone has a fair and just opportunity to attain their optimal health regardless of race, ethnicity, disability, sexual orientation, gender identity, socioeconomic status, geography, preferred language, and other factors that affect access to care and health outcomes [41]. Specialized care centers with amyloid PET imaging and CSF sampling are most often available in urban areas, presenting geographic access barriers for rural populations. Women, Black, and Hispanic patients are disproportionately affected by AD [42]. In a subgroup analysis of the main clinical validity study for the PrecivityAD2 blood test, there were no differences in accuracy noted between those subjects by sex, age (60–69, 70–80, and 80+), or race (White, Black, Non-White) [17]. In this current clinical utility study, 28% of patients were typically under-represented Black, Hispanic, and Asian minorities.

There are several limitations regarding the study design and results of the QUIP II study reported here. First, our study evaluated intended changes to the planned clinical action versus the conducted clinical action. This limitation is a function of the study setting and design, as this study was a real-world evaluation of a BBM in a practice setting where clinicians completed a survey under an IRB exemption determination. Second, our focus on outcomes was limited to the clinician-reported probability of AD diagnosis as well as clinical decision-making and resource utilization, such as AD drug therapy and additional brain amyloid evaluation. While we did not directly measure the effects of this BBM test designed to help clinicians with the evaluation of cognitive impairment on patient outcomes such as improved activities of daily living or memory scales, there are several smaller cohort studies and larger, controlled studies in the literature that show the value of non-pharmacological and pharmacological therapies as outlined above that have shown such a benefit. The goal of the BBM test in this setting is to help ensure that the right patient obtains the right diagnosis at the right time: the effect of such improved diagnostic accuracy leading to improved patient management and improved patient outcomes can thus be extrapolated from the clinical literature.

Furthermore, the study design included a single-arm study cohort and no control group, therefore limiting the ability to establish causality between the APS2 test and changes in AD diagnosis probability, drug therapy, and additional brain amyloid evaluation. In addition, the generalizability of the study results cannot be guaranteed; however, we believe that the lack of variation across study sites with reference to changes in clinician-reported AD probability (*p* = 0.22) as well as the inclusion of 28% traditionally under-represented patients in our analysis provide a solid foundation regarding the robustness of the study findings.

## 5. Conclusions

The results from this study suggest that this BBM test was effectively incorporated into clinical practice with high concordance to the intended use population, thus representing appropriate patient selection by clinicians for this BBM test. Furthermore, the use of the PrecivityAD2 blood test by memory specialists was associated with clinically meaningful changes in decision-making around AD diagnostic certainty, drug therapy management, and additional brain amyloid evaluation among patients evaluated for cognitive impairment. While the clinical utility of CSF biomarkers [43] and PET biomarkers [44,45] have been demonstrated in the literature, there is considerably less evidence of clinical utility with blood biomarkers. Clinical implementation of this BBM test is likely to increase diagnostic certainty and impact clinical management in patients with MCI or dementia by helping clinicians rule in AD and identifying patients who may benefit from DMTs, as well as rule out AD in patients to allow for other diagnostic considerations for their cognitive symptoms. Other potential roles for blood biomarkers for use in clinical practice include screening to detect persons at risk for AD, determination of prognosis and disease progression, and therapeutic drug monitoring. Further research is needed to evaluate the cost impact and economic utility of integrating blood biomarker tests into clinical practice; recent studies have yielded encouraging findings regarding these key themes [46,47].

## Figures and Tables

**Table 1:** Study demographics.

	Summary Statistics
Number of patients	203
Age (years)	
Median (range)	74 (54–90)
Gender, *n* (%)	
Female	108 (53%)
Male	92 (45%)
Did not answer	3 (2%)
Race, *n* (%)	
White	153 (75%)
Black	40 (20%)
Asian	6 (3%)
Did not answer	4 (2%)
Ethnicity, *n* (%)	
Not Hispanic or Latino	188 (93%)
Hispanic or Latino	11 (5%)
Did not answer	4 (2%)

**Table 2:** Changes in medication use from pre- and post-BBM testing.

APS2 Category	Any Medication		*p*-Value	Relative Change from Usual Care
	Pre	Post		
Negative (*n* = 104)	77 (74%)	20 (19%)	7.06 × 10^−15^	74% Decrease
Positive (*n* = 99)	71 (71%)	96 (96%)	2.68 × 10^−6^	35% Increase
Overall (*n* = 203)	148 (72%)	116 (57%)	0.0013	35% Decrease

**Table 3:** Changes in additional brain amyloid evaluation from pre- and post-BBM testing.

APS2 Category	Any Test		*p*-Value	Relative Change from Usual Care
	PRE	POST		
Negative (*n* = 104)	43 (41%)	13 (12%)	5.81 × 10^−6^	70% Decrease
Positive (*n* = 99)	35 (35%)	26 (26%)	0.218	26% Decrease
Overall	78 (38%)	39 (19%)	3.13 × 10^−5^	50% Decrease

## Data Availability

Investigators may request access to anonymized individual patient data and redacted trial documents, including raw datasets, analysis-ready datasets, trial protocols, annotated case report forms, statistical analysis plans, dataset specifications, and clinical trial reports 20 months after the trial is complete. Prior to the use of the data, proposals need to be approved, and a signed data-sharing agreement will then be put in place. All documents are for a predetermined time, typically 12 months.

## Supplementary Materials

The following supporting information can be downloaded at: https://www.mdpi.com/article/10.3390/diagnostics15020167/s1, Table S1: Changes in medication use by medication category.
