# Supplementary material for: Clinical Utility of an Alzheimer’s Disease Blood Test Among Cognitively Impaired Patients: Results from the Quality Improvement PrecivityAD2 (QUIP II) Clinician Survey Study
Source: Diagnostics (Basel). 2025 Jan 13;15(2):167. doi: 10.3390/diagnostics15020167 (PMC11764142; doi:10.3390/diagnostics15020167)
Supplement: Supplementary file 1 [file diagnostics-15-00167-s001.zip › diagnostics-3367812-supplementary.pdf]

Supplementary Table S1. Changes in medication use by medication category

1.1 Cholinesterase Inhibitor

APS2 Category	Cholinesterase Inhibitor Use		P-value	Relative Change from Usual Care
	PRE	POST		
Negative (n=104)	66 (63%)	14 (13%)	3.63e-13	79% Decrease
Positive (n=99)	64 (65%)	87 (88%)	0.000238	36% Increase
Overall	130 (64%)	101 (50%)	0.00502	22% Decrease

1.2 Memantine

APS2 Category	Memantine Use		P-value	Relative Change from Usual Care
	PRE	POST		
Negative (n=104)	41 (39%)	9 (9%)	4.9e-07	78% Decrease
Positive (n=99)	43 (43%)	59 (60%)	0.0329	37% Increase
Overall	84 (41%)	68 (33%)	0.124	19% Decrease

2.4.3 Lecanemab

APS2 Category	Lecanemab Use		P-value	Relative Change from Usual Care
	PRE	POST		
Negative (n=104)	29 (28%)	0 (0%)	2.08e-08	100% Decrease
Positive (n=99)	30 (30%)	47 (47%)	0.0197	57% Increase
Overall	59 (29%)	47 (23%)	0.214	20% Decrease
